# The degeneration and replacement of dopamine cells in Parkinson’s disease: the role of aging

**DOI:** 10.3389/fnana.2014.00080

**Published:** 2014-08-07

**Authors:** Manuel Rodriguez, Ingrid Morales, Clara Rodriguez-Sabate, Alberto Sanchez, Rafael Castro, Jose Miguel Brito, Magdalena Sabate

**Affiliations:** ^1^Laboratory of Neurobiology and Experimental Neurology, Department of Physiology, Faculty of Medicine, University of La LagunaLa Laguna, Tenerife, Canary Islands, Spain; ^2^Center for Networked Biomedical Research in Neurodegenerative Diseases (CIBERNED)Madrid, Spain; ^3^Rehabilitation Service, Department of Physical Medicine and Pharmacology, Faculty of Medicine, University of La LagunaLa Laguna, Tenerife, Canary Islands, Spain

**Keywords:** Parkinson’s disease, aging, dopamine, nigrostriatal cells, etiopathology

## Abstract

Available data show marked similarities for the degeneration of dopamine cells in Parkinson’s disease (PD) and aging. The etio-pathogenic agents involved are very similar in both cases, and include free radicals, different mitochondrial disturbances, alterations of the mitophagy and the ubiquitin-proteasome system. Proteins involved in PD such as *α*-synuclein, UCH-L1, PINK1 or DJ-1, are also involved in aging. The anomalous behavior of astrocytes, microglia and stem cells of the subventricular zone (SVZ) also changes similarly in aging brains and PD. Present data suggest that PD could be the expression of aging on a cell population with high vulnerability to aging. The future knowledge of mechanisms involved in aging could be critical for both understanding the etiology of PD and developing etiologic treatments to prevent the onset of this neurodegenerative illness and to control its progression.

Parkinson’s disease (PD) is a neurodegenerative illness whose onset and progression is clearly linked to aging (Driver et al., 2009; Buchman et al., 2012). The discovery of cell loss and eosiniphilic intracitoplasmic aggregates (Lewy bodies) in the substantia nigra (SN) of these patients during the early twentieth century (Greenfield and Bosanquet, 1953) led a number of groups to investigate the etio-pathology of PD in this center. Although recent studies have reported neurodegeneration in many other brain centers, the degeneration of SN cells is still the hallmark for a diagnosis of PD.

In the 1960s Hornykiewicz reported a decrease of striatal dopamine (DA) and an effective therapeutic response to levodopa (a DA precursor) suggesting that nigrostriatal DA-cells (nsDAc) are the SN cells which mainly degenerate in PD, a possibility also supported by the loss of neuromelanin+ cells in this center (this pigment is a by-product of DA oxidation) (Hornykiewicz, 1966, 2010; Hirsch et al., 1988). This possibility was then supported by studies showing that most degenerated cells express proteins involved in the synthesis (e.g., tyrosine hydroxylase -TH- and l-dopa decarboxylase -DD-), degradation (monoamine oxidase -MAO-), and transport (dopamine transporter, DAT) of DA (Lloyd and Hornykiewicz, 1970; Kastner et al., 1993). The aforementioned findings are frequently used to support the possibility that the nigral DA cell (DAc) loss is a specific characteristic of PD. However, a similar degeneration has been observed in the SN of aged healthy subjects who also show a decrease in the number of: (1) total SN neurons (Hirai, 1968; McGeer et al., 1977; Stark and Pakkenberg, 2004; Morterá and Herculano-Houzel, 2012); (2) pigmented SN neurons (which decrease 7–10% per decade) (Ma et al., 1999; Stark and Pakkenberg, 2004; Rudow et al., 2008); (3) TH+ and DAT+ neurons (Kastner et al., 1993; Rudow et al., 2008; Kordower et al., 2013); (4) DD+ neurons (Lloyd and Hornykiewicz, 1970); and (5) MAO+ neurons (Saura et al., 1997). Thus, the nsDAc loss cannot be considered as a discriminating characteristic of PD.

It has been suggested that the nigral DA-cell subgroups (González-Hernández and Rodriguez, 2000) which degenerate in PD are not the same subgroups which degenerate during the normal aging. PD degeneration mainly affects snDAc located in the ventral tier of the posterior-lateral regions of the SN compacta (Fearnley and Lees, 1991; Damier et al., 1999) which innervate the dorsal-lateral region of the striatum (Kish et al., 1988; Hornykiewicz, 1989). However, this snDAc subgroup also shows the highest degeneration rate during aging, a fact observed in both monkeys (Kanaan et al., 2008; Collier et al., 2011) and humans (Reeve et al., 2014). In addition, the striatal distribution of the DA denervation is also similar in PD (Kish et al., 1988; Hornykiewicz, 1989) and aging (Kish et al., 1992; Haycock et al., 2003). Therefore, the difference between the DA-cell degeneration in PD and aging may be the intensity of the degeneration process more than the type of cells which degenerate.

Both PD (Olanow and Tatton, 1999; Obeso et al., 2010) and aging (Olson, 1987; Peto and Doll, 1997) are probably the consequence of the simultaneous and persistent action of a number of damaging agents, with oxidative stress being one of the most relevant factors in both cases. *Oxidative stress* has proved to be critical for aging (Gerschman et al., 1954; Brack et al., 2000; Toussaint et al., 2000), affecting proteins, lipids, and nucleic acids in a variety of organs and animals (Sohal and Weindruch, 1996; Perez et al., 2009; Oliveira et al., 2010). The oxidative stress in mammals is mainly generated by the mitochondrial production of energy. The nsDAc has an unmielinated axon (Orimo et al., 2011) and a large number of synaptic terminals (hundreds of thousands) (Matsuda et al., 2009) which require a high amount of energy, thereby increasing oxidative stress. The metabolization and autooxidation of DA, together with the high concentration of intracellular iron, are additional sources of free radicals in these cells (Kidd, 2000; Berg and Hochstrasser, 2006). These characteristics increase the vulnerability of the snDAc to the aging process.

The DAc is protected from oxidative stress by different mechanisms including the superoxide dismutase and glutatione peroxidase activity (which prevent the oxidant action of oxygen species), and by the DAT and the vesicular monoamine transporter 2 activity (which moves DA from the extracellular medium to synaptic vesicles preventing its metabolization and self-oxidation). These protecting mechanisms are altered in PD where a disruption of the mitochondrial electron transport chain increases the generation of free radicals (Parker et al., 1989; Bender et al., 2006). This, and the down-regulation of the superoxide dismutase, glutatione peroxidase, DAT and vesicular monoamine transporter 2 activities observed in PD (Riederer et al., 1989; Zeevalk et al., 2008), suggest high oxidative stress in the SN of these patients. This possibility is also supported by the high oxidative damage of lipids (Bosco et al., 2006), proteins and DNA (Nakabeppu et al., 2007) found in the SN of these patients (Jenner, 2007). However, all these facts have also been observed in the aged brain and cannot be considered as a selective characteristic of the PD brain (Sohal and Brunk, 1992; Oliveira et al., 2010). In fact, increasing the resistance to oxidative stress via caloric restriction is often considered as the most effective way of delaying aging in animals (Yu, 1996; Bokov et al., 2004), although this neuroprotecting possibility is still to be properly tested in PD.

The most direct impact of oxidative stress is produced on the *mitochondria*. The DNA of mitochondrias (mtDNA) is highly vulnerable to mutations because it is located near the mitochondrial source of free radicals (electron transport chain) and because it is not protected by histones. mtDNA shows a high number of delections in PD patients, and epidemiological studies and cybrid models have suggested that the mtDNA damage is important in PD (Gu et al., 1998; Kraytsberg et al., 2006). Similar mtDNA damage has been observed in the healthy brain, where the mtDNA mutations normally accumulate with aging (Linnane et al., 1989; Bender et al., 2006). Sporadic mtDNA mutations in single mitochondrias are not enough to induce severe cell damage, but the aggregation of random mutations in an increasing number of mitochondrias can reduce cell viability. This fact probably enhances neurodegeneration in both aged and age-associated diseases such as PD (Cantuti-Castelvetri et al., 2005; Smigrodzki and Khan, 2005; Maruszak et al., 2006).

The mitochondrial population of cells is normally protected from damage by different repair mechanisms, including *fission/fusion processes* (which use healthy mitochondrias to recuperate the functions of damaged mitochondrias) and *mitophagy* (an autophagic process which eliminates the most damaged mitochondrias preventing their accumulation). Proteins involved in these repair mechanisms (e.g., parkin and PINK1) behave anomalously in both PD (Ethell and Fei, 2009) and aging (Palikaras and Tavernarakis, 2012), with autophagy also being altered in both cases (Cuervo et al., 2004; Ethell and Fei, 2009; Hubbard et al., 2012). The movement of mitochondrias across the axon is necessary to preserve an efficient quality control of neuronal mitochondrias. Most synaptic mitochondrias are synthesized in the neuronal somata and moved along axons (anterograde motion). Axonal transport is also necessary to move dysfunctional mitochondrias from synaptic bottoms to the cell somata (retrograde motion) where they can be destroyed by mitophagy and other mechanisms (Cheng et al., 2010). Different proteins involved in the axonal transport (e.g., α-synuclein, parkin and PINK1-Miro-Milton complex) are involved in both PD and aging as well. The axonal damage observed in DAc of the PD brain (Cheng et al., 2010) has been found in the aging brain too (Gilley et al., 2012), which shows that the anomalous behavior of axons is also a characteristic shared by the PD and the aging brain.

The anomalous conformations of α-synuclein facilitate the formation of Lewy bodies in the nsDAc of PD patients (Lansbury and Brice, 2002) as well as in healthy aged subjects (Li et al., 2004; Moore et al., 2005). Similarly, the mutation of parkin has been associated to both PD (Lücking et al., 1998; Lucking et al., 2000; Moore et al., 2005; Reeve et al., 2014) and aging (Rodríguez-Navarro et al., 2007; Vincow et al., 2013). The UCH-L1 mutation impairs the ubiquitin-proteasome system (Osaka et al., 2003; Li et al., 2004), promoting both PD (Leroy et al., 1998) and aging (Marzban et al., 2002). PINK1 facilitates axonal transport and degradation of damaged mitochondrias (Valente et al., 2004a; Liu, 2014), and this PINK1 activity is altered in both the PD (Valente et al., 2004b; Albanese et al., 2005; Gelmetti et al., 2008) and aging (Wood-Kaczmar et al., 2008; Vincow et al., 2013) brain. The DJ-1 protein protects cells against oxidative stressors (Moore et al., 2005). Its anomalous behavior has been linked to a familiar parkinsonism (Bonifati et al., 2003a,b; Ibanez et al., 2003) and to aging (Marzban et al., 2002; Meulener et al., 2006). These proteins have been associated with the different familiar early onset parkinsonisms which present mutations of their genes, but also with idiopathic (or sporadic) PD and with normal aging where their activity may change (Cookson and Bandmann, 2010).

Many of the altered cell groups in PD show similar changes in the aged brain. This is the case of *astrocytes*, cells whose physiological functions (Sofroniew and Vinters, 2010; Rodriguez et al., 2012) change in PD and aging (Raivich et al., 1999; Morales et al., 2013). Astrocytes prevent neuronal damage by releasing neuroprotecting agents (glutathione, basic fibroblast growth factor, glial cell line-derived neurotrophic factor…) (Saavedra et al., 2006; Deierborg et al., 2008), and by removing toxic molecules from the extracellular medium (e.g., *α*-synuclein) (Braak et al., 2007; Lee et al., 2010). The neuroprotecting abilities of astrocytes decrease with age (Pertusa et al., 2007; Mansour et al., 2008; Chinta et al., 2013), which increases DAc vulnerability (Mirza et al., 2000; Song et al., 2009) and enhances the development of PD (Halliday and Stevens, 2011).

It has been suggested that the slow DAc decline during life is normally compensated by a slow cell repopulation provided by the subventricular zone (SVZ; Doetsch et al., 1997, 1999; Quiñones-Hinojosa et al., 2006). SVZ *stem cells* normally differentiate into astrocytes and neuroblasts which later migrate to the olfactory bulb. The differentiation and migration of these cells are modulated by the DA released from nsDAc terminals (Freundlieb et al., 2006; Borta and Höglinger, 2007). Some neurons generated by SVZ stem cells express a DAergic phenotype and migrate to the olfactory bulb where they modulate olfaction. However, neuroblasts can also migrate to other brain loci, particularly when the target areas have been damaged (ictus‥) (Macas et al., 2006). It has been suggested that stem cells can migrate to the SN (Kay and Blum, 2000; Zhao et al., 2003; Zhao and Janson Lang, 2009), where they could compensate for the DAc loss induced by aging. Thus, an insufficient repopulation of the DAc loss induced by senescence may also be a cause of PD (Armstrong and Barker, 2001). This possibility is supported by the low neurogenesis observed in the SVZ (Höglinger et al., 2004) and anterior olfactory nucleus (Pearce et al., 1995; Hawkes et al., 1997) of PD patients. A similar low neurogenesis has been observed during aging. Healthy subjects present a noticeable decrease of SVZ stem cell proliferation during the last third of life which is when the incidence of PD increases (Galvan and Jin, 2007; Conover and Shook, 2011). Nevertheless, the cell repopulation hypothesis is currently a matter of debate because the DAergic repopulation of the SN has not been definitively proved (Frielingsdorf et al., 2004). The new astrocytes derived from SVZ stem cells could also prevent DAc degeneration by replacing the damaged astrocytes in PD patients (Gonzalez-Perez and Quinones-Hinojosa, 2012; Mack and Wolburg, 2013). Bearing in mind the neuroprotecting role of astrocytes, this repopulation could also be necessary to keep the DAc alive in the aged brain. In this case, aging and PD could be the final result of a deficient gliogenesis and of the consequent deterioration of the astrocyte population supporting the snDAc. Therefore, the reduced neurogenesis and gliogenesis secondary to the senescence of the SVZ could be involved in both aging and PD.

The *microglia* has been linked to the neurodegenerating process in PD. *Microglia* is activated in the presence of aggregated forms of α-synuclein (Zhang et al., 2005), expressing macrophage markers and releasing IL-1β, IL-6 and TNF-α which can damage the DAc (Croisier et al., 2005; Orr et al., 2005). This activation has been found in both PD (Hunot et al., 1996; Knott et al., 2000) and aged brains (Godbout and Johnson, 2004; Gelinas and McLaurin, 2005; Campuzano et al., 2009), suggesting that the neurotoxic action of these cells is similar in both conditions (Ouchi et al., 2005; Streit et al., 2008; Cunningham, 2013).

Recent technological advances have made it possible to obtain *pluripotent stem cells* (iPSC; Takahashi and Yamanaka, 2006) from the skin of healthy subjects and patients with different illnesses including PD (disease-specific iPSC) (Lee and Studer, 2010). The DAc derived from iPSC shows an abnormal phenotype (with respect to aged-matched controls) when produced from patients with familiar parkinsonisms (PINK1, SNCA, parkin, LRRK2…) (Sánchez-Danés et al., 2013) but not when produced from patients with sporadic PD (Soldner et al., 2009). However, cells from sporadic PD patients show the typical alterations of the nsDAc when they are kept for a long time in a culture medium which *in vitro* simulates *in vivo* aging (more than 2 months in a culture medium which induces chronic cellular stress) (Sánchez-Danés et al., 2012). In these conditions, the DAc derived from iPSC of sporadic PD patients shows morphological (reduced number of neurites and accumulation of autophagic vacuoles) and neurochemical (accumulation of α-synuclein in their cytoplasm) characteristics similar to those of the DAc in PD (Sánchez-Danés et al., 2012). Thus, aging, in this *in vitro* model, seems to be a condition for developing the DAc characteristics observed in PD, which also supports aging as a basic mechanism for PD.

In summary, the studies reviewed above show that the DAc degeneration in PD is similar to that observed in aging, suggesting that aging is not simply another agent to add to the etiology of PD. The progressive course of aging and PD could be induced by the same multi-factorial etiology, including astrocytic and microglia alterations, oxidative stress, anomalous action of different proteins, mitochondrial disturbances, and alterations of the mitophagy and the ubiquitin-proteasome system. To this effect, PD could be the expression of aging on a cell population which, due to its characteristics (number of synaptic terminals, unmielinated axon etc…), is particularly vulnerable to damage. Repeated injuries accumulated throughout a person’s lifespan may go unnoticed until the DAc loss exceeds a critical value. DAc degenerated over the years could be regularly replaced by new neurons derived from brain stem cells. Since stem cells are also affected by aging, the DAc loss induced by aging could be increased by an insufficient cell replacement. The progressive imbalance between the DAc loss and DAc neurogenesis eventually leads to a large enough decrease in the number of DAc to trigger the onset of motor disturbances of PD. This DAc loss is usually considered as a sign of brain aging until it crosses the above mentioned clinical threshold and PD can be diagnosed. In our opinion, a better understanding of the mechanisms involved in aging would help to explain the etio-pathology of PD.

## Conflict of interest statement

The authors declare that the research was conducted in the absence of any commercial or financial relationships that could be construed as a potential conflict of interest.
